# Altered Cognitive Networks Connectivity in Parkinson's Disease During the Microlesion Period After Deep Brain Stimulation

**DOI:** 10.1111/cns.70184

**Published:** 2024-12-25

**Authors:** Bei Luo, Yanxiang Zou, Jiuqi Yan, Jian Sun, Xiang Wei, Lei Chang, Yue Lu, Liang Zhao, Wenwen Dong, Chang Qiu, Jun Yan, Yanhong Zhang, Wenbin Zhang

**Affiliations:** ^1^ Department of Functional Neurosurgery The Affiliated Brain Hospital of Nanjing Medical University Nanjing China; ^2^ School of Nursing Nanjing Medical University Nanjing China; ^3^ Department of Geriatric Neurology The Affiliated Brain Hospital of Nanjing Medical University Nanjing China; ^4^ The Affiliated Brain Hospital of Nanjing Medical University Nanjing China

**Keywords:** deep brain stimulation, functional magnetic resonance imaging, microlesion effect, Parkinson's disease, resting‐state functional connectivity, verbal fluency

## Abstract

**Aims:**

Cognitive functions are reduced in Parkinson's disease (PD) patients after deep brain stimulation (DBS) surgery. However, the underlying mechanisms remain unclear. The current study attempted to elucidate whether DBS alters the functional connectivity (FC) pattern of cognitive networks in PD patients.

**Methods:**

The study obtained fMRI and cognitive scale data from 37 PD patients before and after the DBS surgery. Seed‐based FC analysis helped demonstrate the FC changes of the default mode network (DMN), executive control network (ECN), and dorsal attention network (DAN).

**Results:**

PD patients indicated significant network connectivity decline in DMN [such as in right precuneus, left angular gyrus, and left middle frontal gyrus (MFG)], ECN [such as in left inferior parietal gyrus, left MFG, and left supplementary motor area (SMA)], and DAN [such as in left inferior frontal gyrus and left MFG] post‐DBS surgery. The phonemic fluency score was positively associated with the FC value of the right precuneus and left angular gyrus in DMN before DBS.

**Conclusion:**

The general reduction in FC in the major cognitive networks after DBS surgery depicted the presence of the corresponding network reorganization. Further research can help explore the mechanism of impaired cognitive function post‐DBS.

## Graphical abstract

Seeds‐based functional connectivity analysis revealed significant alterations in cognitive networks during microlesion period after deep brain stimulation. Functional connectivity within the three major networks of the default mode network, executive control network, and dorsal attention network showed a widespread decline after surgery.

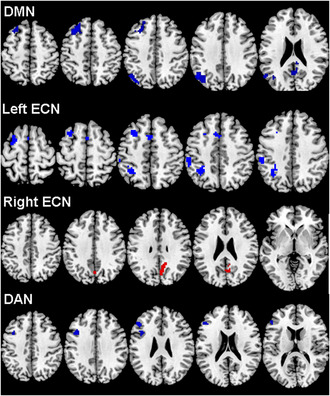

## 1 Introduction

Deep brain stimulation (DBS) is an effective surgical treatment in Parkinson's disease (PD) patients [1, 2]. DBS is gradually replacing lesioning surgery because of its reversibility, adjustability, safety, and effectiveness, and it is becoming increasingly widely used. DBS plays a therapeutic role by inserting a pulse transmitter that emits current to control neural circuits or networks [3, 4]. Immediate improvement in motor symptoms could be observed after DBS (off‐stimulation) among PD patients. This phenomenon is called the microlesion effect (MLE) [5, 6]. This occurs after the electrodes are implanted within the subthalamic nucleus (STN) before active stimulation. Despite being common in clinical practice, the current mechanism remains unclear. Previous studies have established its association with the brain tissue lesion induced by the electrode track, local neuron destruction to release neurotransmitters, and post‐injury edema around the electrode track [6, 7]. The efficacy of DBS on motor symptoms of movement disorders is widely known. However, the cognitive side effects of STN‐DBS on PD patients have attracted increased attention [8]. Studies have observed that cognitive function, especially verbal fluency (VF), is decreased in PD patients after DBS [9, 10]. Previous studies have observed that VF was not affected by stimulation, but it has declined post‐DBS surgery [11, 12]. The MLE effect could be the primary reason for reduced VF after DBS.

Functional magnetic resonance imaging (fMRI) has been a widely used non‐invasive method to determine regional and global brain connectivity possessing high spatiotemporal resolution [13, 14]. Resting‐state functional connectivity (FC) indicates synchronized neural activity and is affected by neuropathological and neurochemical changes in degenerative diseases [15, 16]. Therefore, resting‐state FC analysis could help identify functionally integrated brain regions linked with pathological changes during neurodegenerative disorders. Due to dopamine depletion induced by degenerative changes in dopamine neurons inside the substantia nigra of the midbrain, PD patients demonstrate reorganized and striatal‐cortical functional networks [17, 18, 19, 20]. Several studies have revealed altered FC in PD patients with mild cognitive impairment [21, 22, 23]. Some parameters can become markers to identify different cognitive impairment subtypes [24, 25]. However, it is unclear whether cognitive impairment in PD patients during the microlesion phase post‐DBS is associated with cognitive network pattern disruption. Previously, we used regional homogeneity and low‐frequency fluctuation methods to analyze changes in brain activity before and after DBS surgery [26, 27]. The results indicated that the activity in many brain areas was altered after DBS surgery [26, 27]. Understanding changes in brain FC during the microlesion period after DBS surgery could be a promising approach to deciphering the underlying mechanisms of these clinical changes in cognitive function decline.

The current study focused on the effects of DBS surgery on cognition, such as language function, in PD patients. No research has been conducted to date to explore the impact of DBS on large‐scale cognitive networks. Therefore, this prospective study was to better characterize system‐level changes in resting‐state FC during the microlesion period post‐DBS in PD patients. Functional networks previously implicated in PD were selected for seed‐based analysis: default mode network (DMN), executive control network (ECN), and dorsal attention network (DAN). These networks are related to memory, attention, executive, and cognitive functions [20, 21, 28]. Exploratory analyses helped investigate the correlation between FC alterations and clinical symptom changes.

## 2 Materials and Methods

### 2.1 Participants

Initially, 44 PD patients were recruited for this study. PD patients were diagnosed according to the United Kingdom Parkinson's Disease Society Brain Bank clinical diagnostic criteria [29] and the MDS clinical diagnostic criteria for PD [30]. All the patients satisfied the indications and underwent DBS surgery. Patients with central nervous system disease, drugs affecting brain function (antipsychotics), significant cognitive impairment, co‐existing medical conditions affecting surgery or survival, or any MRI contraindications were excluded. The hospital ethics committee approved the study, and all the patients signed written informed consent before participating in the experiment.

### 2.2 Clinical Assessment

The Montreal Cognitive Assessment (MoCA) assesses the cognitive level of patients. Their mental state was assessed with the Hamilton Anxiety Rating Scale (HAMA) and the Hamilton Depression Rating Scale (HAMD). The VF test, including semantic and phonemic fluency, determines verbal memory and spontaneous language ability. Considering the cultural background of patients, the Chinese version of the phonemic fluency test was used for assessment. Three Chinese characters (“小”, “天”, and “大”) were presented individually, each lasting 20 s, and the patients had to create as many different phrases or 4‐character idioms as possible within 60 s. The semantic fluency test requires participants to name as many animals as possible in 60 s. The VF score is the number of animal names or words spoken in a minute. If the data collected was closer to the surgery date, some potential time implications could be avoided. Therefore, data acquisition was performed 3 days before and 1 day after surgery. Since the MLE disappeared 1 month after surgery, data was collected after 1 month of DBS. For the MoCA and VF, PD patients were assessed thrice: 3 days before DBS, 1 day after DBS, and 1 month after DBS. The data from 3 days before DBS and 1 day after DBS were divided into PD‐Pre‐DBS and PD‐Post‐DBS groups. All the scale and MRI data were obtained at least 12 h after discontinuing antiparkinsonian drugs to reduce the drug impact on data collection.

### 2.3 Surgery

The bilateral STN was chosen as the target for surgical implantation among all PD patients. The preoperative brain MRI and skull CT with frame data could be imported into the surgery planning software. The midpoint of the anterior and posterior commissures became the origin points for developing a spatial coordinate system. The STN target is positioned 11–12 mm beside the origin, 3 mm backward, and 4 mm downward. Moreover, the target coordinates were fine‐tuned based on the specific STN nuclei location in the MRI images. The implantation path was designed prior to the coronary suture, avoiding the ventricles, sulci, and cortical vessels. Under local anesthesia, the electrodes were implanted, while the pulse emitter was implanted under general anesthesia. The patient's head CT was reviewed 1 week after the operation, and the fusion CT with the preoperative MRI image data revealed an accurate electrode implantation position.

### 2.4 Image Data Acquisition

Structural and functional imaging data were obtained from all the participants on a 1.5 T MRI scanner (GE Medical System) equipped with an 8‐channel head coil. The participants were asked to remain calm, close their eyes, stay awake, and not think about anything specific while scanning. Functional images were obtained using an echo planar imaging (EPI) sequence using the following parameters: repetition time (TR) = 2000 ms, echo time (TE) = 40 ms, flip angle (FA) = 90°, thickness = 3.0 mm with no gap, number of slices = 28, matrix size = 64 × 64, field of view (FOV) = 240 mm × 240 mm, voxel size = 3.75 × 3.75 × 3 mm^3^, and total number of volumes = 128. The structural images were scanned using a 3D magnetization‐prepared rapid gradient‐echo (MPRAGE) sequence using the following parameters: TR = 11.864 ms, TE = 4.932 ms, FA = 20°, thickness = 1.4 mm, number of slices = 112, matrix size = 256 × 256, FOV = 152 mm × 152 mm, voxel size = 0.59 × 0.59 × 1.4 mm^3^.

### 2.5 Data Preprocessing

Functional data were preprocessed using the resting‐state fMRI data processing assistant (DPABI_7.0, http://rfmri.org/DPABI) [31] with the MATLAB 2013b platform (https://www.mathworks.com/products/matlab), according to the previously published preprocessing steps [20, 32, 33]. The first five time points were removed, followed by slice‐time and motion corrections. The remaining images were normalized to the Montreal Neurological Institute (MNI) space and resampled to a voxel size of 3 × 3 × 3 mm^3^. Then, the data were spatially smoothed using a Gaussian kernel with full width at half maximum of 4 mm × 4 mm × 4 mm. General linear model regression removed interfering variables, including the Friston‐24 parameter, white matter signals, cerebrospinal fluid signals, and linear trends. Later, a time‐bandpass filter (0.01 Hz < *f* < 0.10 Hz) helped eliminate the influence of high‐frequency physiological and low‐frequency drift noise. Participants were excluded if their heads translated more than 3.0 mm or rotated more than 3.0°. Around seven participants were excluded because of excessive head movement.

### 2.6 Functional Connection Analysis

A seed‐based approach helped study the resting‐state cognitive network patterns in DMN, ECN, and DAN. Due to the resampling voxel size of 3 × 3 × 3 mm^3^, the 6 mm radius of the spherical regions of interest (ROIs) becomes a compromise between avoiding an overlap of functionally different brain regions within a single seed region and including multiple voxels within the ROIs to represent cluster activation rather than single voxel activation [32]. Therefore, the ROIs were defined as a sphere with a 6 mm radius around the MNI coordinates. The MNI coordinates for each resting‐state network of the corresponding ROIs were DMN [posterior cingulate cortex (PCC), *x*/*y*/*z* = 1/−55/17], ECN [left and right dorsolateral prefrontal cortex (DLPFC), *x*/*y*/*z* = −42/34/20 and 44/36/20], and DAN [left and right intraparietal sulcus/superior parietal lobe (IPS/SPL), *x*/*y*/*z* = −25/−53/52 and 25/−57/52]. The coordinates of the above seeds were obtained from previous studies [32, 34]. The time series within each ROI was averaged, and its association with every other voxel in the brain was tested with Pearson's correlation coefficient to obtain the FC maps. Finally, the obtained FC maps were converted to a *z*‐value using Fisher's *z*‐transformation for further statistical analysis.

### 2.7 Statistical Analysis

Repeated analysis of variance measures helped statistically analyze clinical scales in PD patients with the SPSS22 software (IBM Corp.), with *p* < 0.05 statistically significant. A paired *t*‐test helped detect the difference in FC before and after DBS surgery among 37 PD patients, with the average frame displacement value used as a covariate. The following thresholds were applied for these voxel statistics: uncorrected height threshold (*p*‐value < 0.001) and family‐wise error rate‐corrected cluster extent threshold (*p*‐value < 0.05). The study determined the correlations between brain network connectivity and clinical variables in PD patients. The FC values of brain areas differing before and after surgery were extracted for each network. The FC value correlation was measured with preoperative scores, such as MoCA, HAMA, HAMD, and VF. We also investigated whether changes in FC values before and after DBS surgery were associated with decreased MoCA and VF scores. The above analysis was performed with Pearson's correlation using the SPSS 22.0 software.

## 3 Results

### 3.1 Demographic and Clinical Characteristics

The demographic characteristics of PD patients are depicted in Table 1. After 1 day of DBS, the MoCA (*p* < 0.001), phonemic fluency (*p* < 0.001), and semantic fluency (*p* < 0.001) scores of PD patients decreased significantly. After 1 month of DBS, MoCA (*p* = 0.015), phonemic (*p* < 0.001), and semantic (*p* < 0.001) scores became lower than the preoperative level.

**TABLE 1:** Demographic and clinical data of all subjects.

	PD (*n* = 37) Mean ± SD	*p*
Age (years)	61.46 ± 9.47	—
Sex (male/female)	17/20	—
HAMA	6.43 ± 3.93	—
HAMD	6.49 ± 4.13	—
MoCA		
3 days before DBS	24.65 ± 2.65	< 0.001^a^, *
1 day after DBS	22.11 ± 4.40	< 0.001^b^, *
1 month after DBS	24.08 ± 3.00	0.015^c^, *
Semantic fluency		
3 days before DBS	19.97 ± 4.22	< 0.001^a^, *
1 day after DBS	13.78 ± 3.94	< 0.001^b^, *
1 month after DBS	16.35 ± 3.81	< 0.001^c^, *
Phonemic fluency		
3 days before DBS	10.08 ± 1.95	< 0.001^a^, *
1 day after DBS	7.49 ± 1.71	< 0.001^b^, *
1 month after DBS	8.78 ± 1.92	< 0.001^c^, *

Abbreviations: DBS, deep brain stimulation; HAMA, Hamilton Anxiety; HAMD, Hamilton Depression; MoCA, Montreal Cognitive Assessment; Mean ± SD, mean ± standard deviation; PD, Parkinson's disease.

^a^ Repeated measures ANOVA test.

^b^ Paired *t*‐test (3 days before DBS vs. 1 day after DBS).

^c^ Paired *t*‐test (3 days before DBS vs. 1 month after DBS).

* *p* < 0.05.

### 3.2 Altered Network Connectivity

**FIGURE 1:**
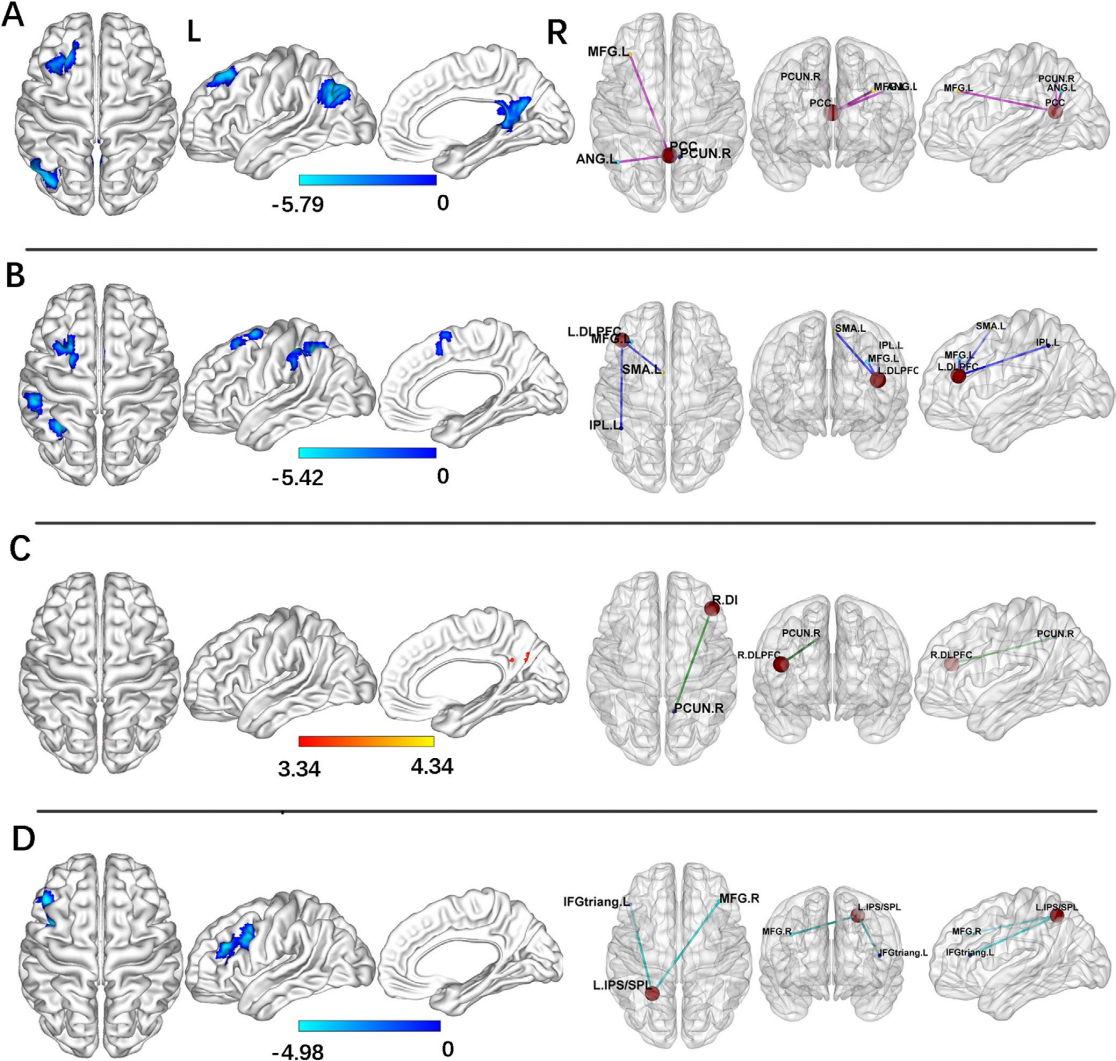
Differences in brain functional connectivity in PD patients before and after DBS surgery (voxel *p* < 0.001, FWE correction with cluster *p* < 0.05). PCC (A), left DLPFC (B), right DLPFC (C), and left IPS/SPL (D) for seeds‐based FC analysis; L, left hemisphere and R, right hemispheres; PCC, posterior cingulate cortex; DLPFC, dorsolateral prefrontal cortex; IPS/SPL, intraparietal sulcus/superior parietal lobe.

**FIGURE 2:**
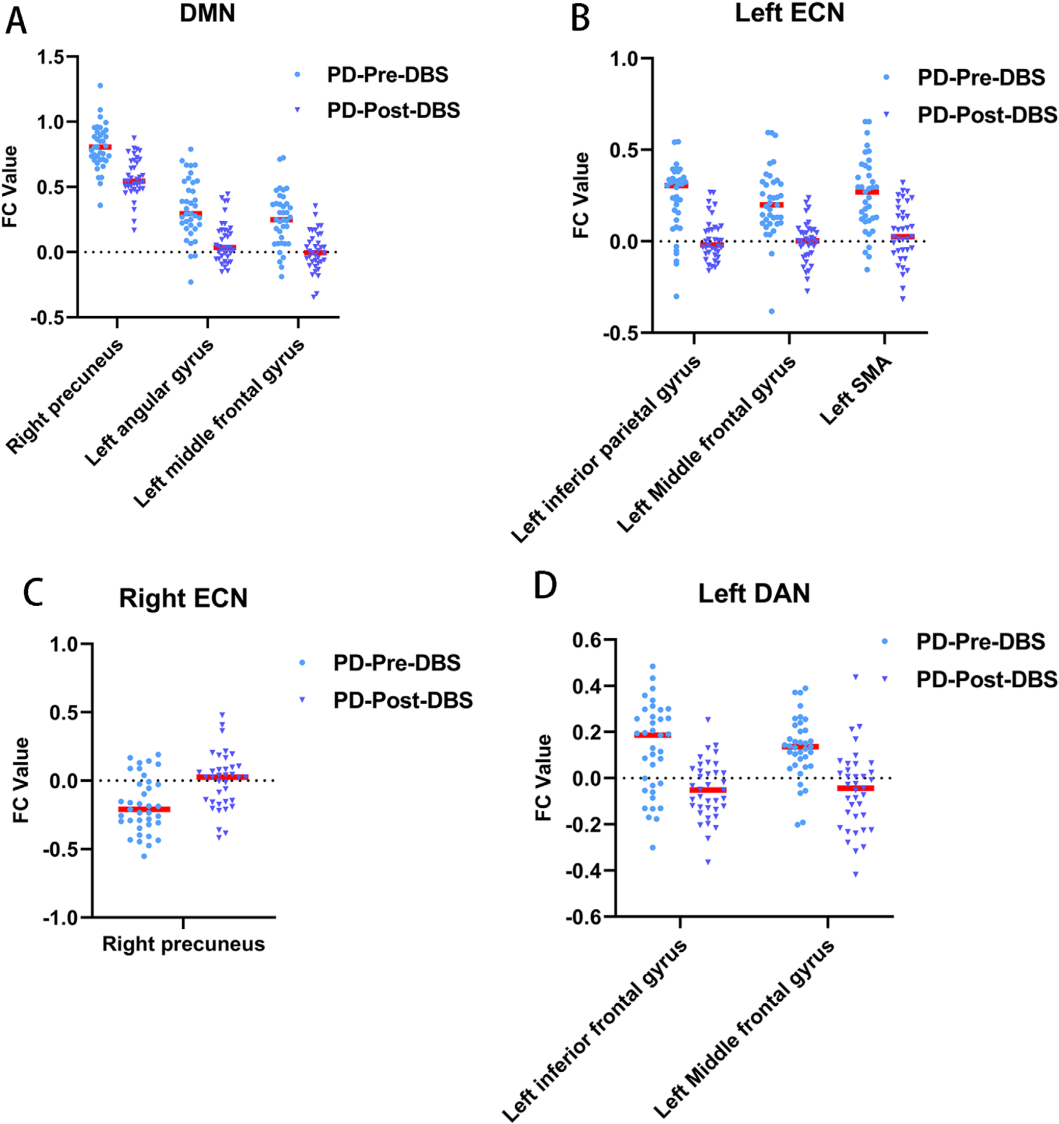
FC values of brain regions with significant differences in the brain network before and after DBS. Posterior cingulate cortex (A), left dorsolateral prefrontal cortex (B), right dorsolateral prefrontal cortex (C), and left intraparietal sulcus/superior parietal lobe (D) were selected for seed‐based analysis.

**FIGURE 3:**
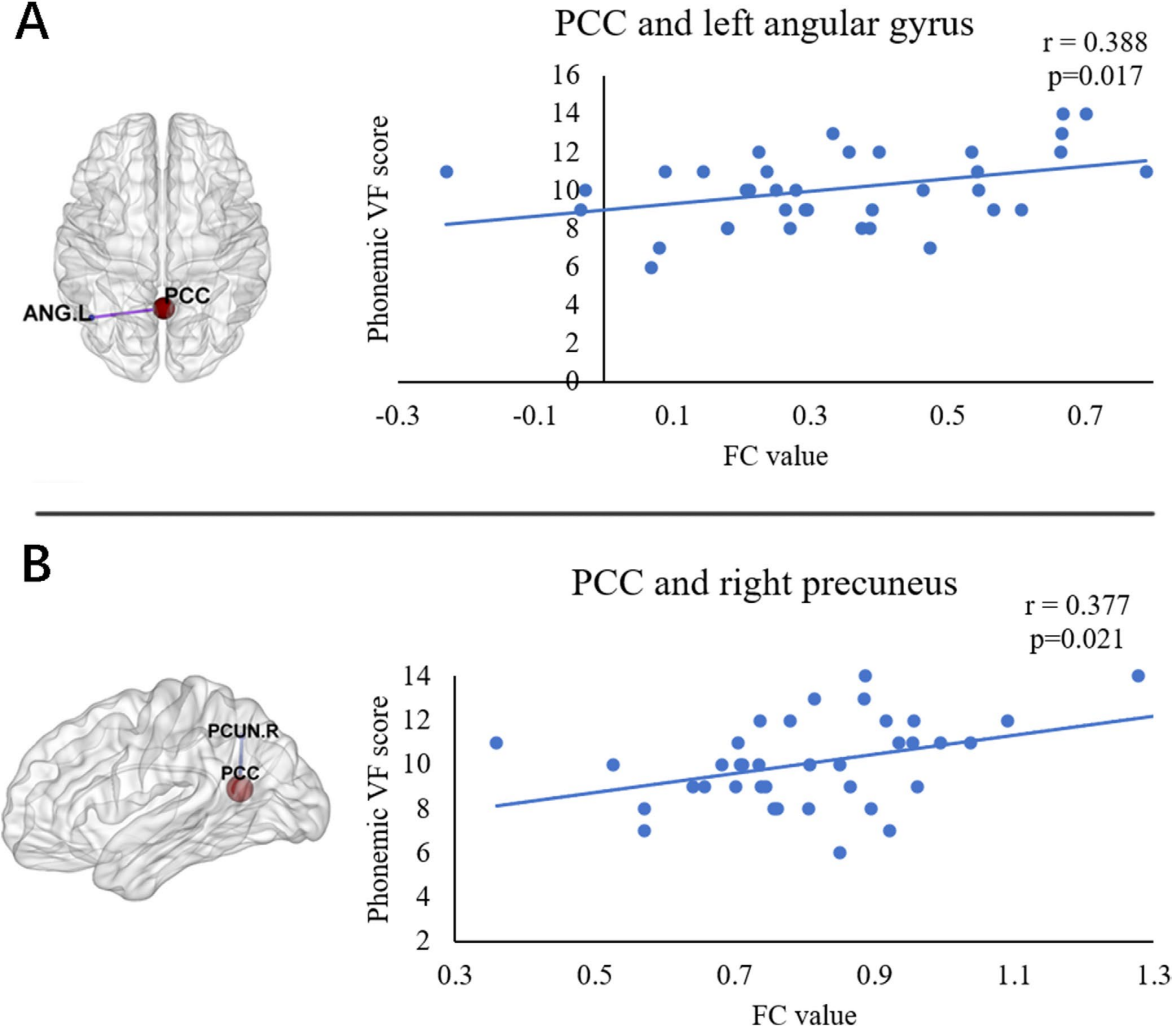
Correlation analysis between brain network FC values and phonemic fluency scores in PD patients before DBS. PCC, posterior cingulate cortex; relationships between: (A) phonemic fluency and FC value of left angular gyrus in DMN, (B) phonemic fluency and FC value of right precuneus in DMN.

For the PCC seed, the PD‐Post‐DBS group had decreased FC in the right precuneus, left angular gyrus, and left middle frontal gyrus (MFG) compared to the PD‐Pre‐DBS group. For the left DLPFC seed, the PD‐Post‐DBS showed significant FC reduction in the left inferior parietal gyrus (excluding supramarginal and angular gyri), left MFG, and left supplementary motor area (SMA) compared to the PD‐Pre‐DBS group. Underconnectivity in the PD‐Post‐DBS group was observed in the triangular part of the left inferior frontal gyrus and left MFG for the left IPS/SPL seed. In addition, PD patients exhibited elevated right DLPFC FC in the right precuneus post‐DBS surgery in ECN. The seed‐based FC analyses are shown in Table 2 and Figure 1. Scatter plots helped depict the FC values of brain regions with significant differences within the brain network before and after surgery (Figure 2). The phonemic fluency score evaluated the correlation analysis, indicating that the spontaneous language ability was positively correlated with the FC value of the right precuneus (*r* = 0.377, *p* = 0.021) and left angular gyrus (*r* = 0.388, *p* = 0.017) in DMN before DBS (Figure 3). Regrettably, no alterations in FC values could correlate with clinical scale changes in the resting‐state network before and after DBS surgery.

**TABLE 2:** Resting‐state functional network alteration comparison between the PD‐Pre‐DBS and PD‐Post‐DBS group.

Seed area	Brain region (AAL)	Cluster size	MNI coordinate (*X*, *Y*, *Z*)			Peak intensity
PD‐Pre‐DBS > PD‐Post‐DBS						
PCC						
Cluster 1	Right precuneus	100	9	−48	12	−4.9166
Cluster 2	Left angular gyrus	193	−48	−63	36	−5.7895
Cluster 3	Left middle frontal gyrus	120	−27	27	45	−5.2425
Left DLPFC						
Cluster 1	Left inferior parietal gyrus	130	−33	−51	48	−4.8716
Cluster 2	Left middle frontal gyrus	86	−33	18	54	−4.8964
Cluster 3	Left SMA	44	−6	15	45	−4.2298
Left IPS/SPL						
Cluster 1	Left inferior frontal gyrus	57	−45	27	30	−4.9773
Cluster 2	Left middle frontal gyrus	59	−39	12	36	−4.3046
PD‐Pre‐DBS < PD‐Post‐DBS						
Right DLPFC						
Cluster 1	Right precuneus	69	9	−63	27	4.3382

Abbreviations: AAL, anatomical automatic labeling; DBS, deep brain stimulation; DLPFC, dorsolateral prefrontal cortex; IPS/SPL, intraparietal sulcus/superior parietal lobe; PCC, posterior cingulate cortex; PD, Parkinson's disease; PD‐Pre‐DBS, 3 days before DBS; PD‐Post‐DBS, 1 day post‐DBS.

## 4 Discussion

The current study provides new information on the relationship between cognitive decline and resting‐state FC patterns during the microlesion phase after DBS. Our results indicated significant changes in major large‐scale cognitive networks after DBS surgery. FC within the three major networks of DMN, ECN, and DAN showed a widespread decline after DBS surgery. Moreover, the left angular gyrus and right precuneus FC value in the DMN were positively associated with the phonemic fluency score before surgery. Therefore, postoperative cognitive changes in PD patients could be related to functional network changes caused by electrode implantation. This highlights the potential network functional reorganization in PD patients post‐DBS.

A slight reduction in cognitive ability can significantly affect quality of life. The growing indications for DBS surgery, the expansion of surgical goals, increasing device sophistication, and recent alterations in stimulation paradigms have enhanced the need to reassess DBS‐related cognitive outcomes [35]. After STN‐DBS, attention, executive functions, VF, and overall cognitive ability were slightly reduced. However, globus pallidus internus (GPi)‐DBS induced less neurocognitive decline than STN‐DBS, such as attention and VF [36]. STN has a regulatory effect on movement, cognition, and emotion. The STN is divided into three functional subregions along its long axis, overlapping in small parts [37, 38]. Stimulating the most dorsal “motor” subregion of STN can improve the pathological network oscillations that induce motor symptoms of PD [39]. However, electrode implantation or clinical stimulation can impact non‐motor subregions and surrounding STN structures. This could explain the greater cognitive and emotional effects of STN‐DBS than GPi‐DBS. In humans, the STN is monosynaptic connected to cognitive brain areas, including the prefrontal cortex. These signals along the hyperdirect pathway can regulate cognitive control in humans. The direct, indirect, and hyperdirect STN pathways are related to several cognitively relevant brain regions, including key brain regions of DMN, ECN, and DAN.

A randomized trial with a delayed stimulation design comparing the cognitive patient status of turned on and not after DBS identified that VF decreased considerably 3 months post‐surgery in both groups compared to the preoperative ones [40]. Thus, cognitive decline was surgery‐induced rather than stimulus‐related [40]. Witt et al. analyzed data from 31 STN‐DBS PD patients and 31 optimal medical treatment groups [41]. The results indicated that VF was impaired in the STN‐DBS group compared with the optimal medical treatment group after 6 months of surgery [41]. The authors analyzed the cortical entry points and electrode trajectories of implanted electrodes. They found that electrode trajectories intersecting with the caudate nucleus elevated the risk of overall cognitive and working memory performance decline [41]. Multiple studies have deciphered that cognitive decline post‐DBS, such as VF, was mainly associated with the electrode trajectory [10, 42]. Subcortical edema induced by electrode implantation could be involved in the transient cognitive decline of PD patients post‐DBS [43]. The current DBS parameters primarily utilize high frequency to treat the motor symptoms linked to PD. Growing evidence depicts that low‐frequency stimulation within STN can regulate cognition [44]. Darrin et al. observed that acute left dorsal low‐frequency STN stimulation enhanced overall speech fluency compared to no stimulation along with dorsal or ventral high‐frequency stimulation [45].

The current study observed that DMN function in PD patients after DBS was severely damaged. There was reduced connectivity in the key centers, including the right precuneus and left angular gyrus. These changes were associated with VF scores, supporting the critical pathophysiological roles. The DMN is vital in cognitive operations linked with social cognition, episodic and autobiographical memory, and language and semantic memory [46, 47]. Due to the critical role of DMN in cognition, impaired DMN function after DBS could be the pathological reason behind cognitive decline in PD patients. Reduced FC between the PCC seed and the left MFG was also observed. Left MFG is linked with higher cognitive control [48], and the cognitive processes are specifically developed in humans [49]. Schmidt et al. [50] assessed VF scores in 85 patients with left middle cerebral artery ischemic stroke and identified that left frontal lobe damage led to significant phonemic fluency impairment. Changes in FC in the left MFG may also be linked to the decline in cognitive tasks, including VF in PD patients post‐DBS surgery. The study did not directly evaluate whether activity in one brain region induced changes in another. Tan et al. observed that the effective connectivity of developmental dyslexia patients reduced from the left precentral gyrus to the right precuneus and angular and from the left MFG to the left calcarine. However, no significant causal relationship could be observed in the cognitive network of the brain regions [51].

The FC of the left inferior parietal gyrus, left MFG, and left SMA within the ECN network was reduced. ECN, often described as a task‐positive network, is associated with executing tasks with higher cognitive requirements [52]. The left inferior parietal gyrus and left MFG are essential brain areas of the ECN. Their decline may indicate a disorder worsening of the postoperative ECN network, affecting the patient's cognitive function. Huang et al. [53] observed that droolers had significantly increased effective connectivity from the right caudate nucleus to the right middle temporal gyrus and inferior parietal lobe compared with PD patients without drooling. The SMA function is related to movement planning. Research suggests that SMA is involved in memory, attention, and executive functions, as well as updating speech functions and spatial information [54, 55]. A study described that a patient who suffered from left anterior cerebral artery hemorrhage leading to reduced function of the left SMA showed residual symptoms of dysfluent halting speech [56]. An 8‐year‐old boy underwent a surgical resection for focal cortical dysplasia in the left SMA, causing impaired speech and executive function [57]. Therefore, decreased FC of SMA could also impair postoperative cognition, particularly VF. The increase in right precuneus FC in the ECN network post‐DBS may compensate for cognitive decline.

The DAN provides top‐down attentional orientation and participates in cognitive training that controls sensory processing [58]. The small‐world organization of DAN benefits general intelligence, verbal intelligence, perceptual reasoning, and executive functioning [59]. Our study observed that the FC of the triangular part of the left inferior frontal gyrus and left MFG decreased in the DAN network. The triangular part of the left inferior frontal gyrus has the most robust regulation of language needs [60, 61, 62]. Compared with typically developing children, the developmental dyslexia group has increased EC from the right cuneus to the left inferior frontal gyrus triangular part [51]. The MFG is the convergence point of dorsal and ventral attentional networks, acting as a network switch interrupting endogenous attentional processes within the DAN [54, 63, 64]. Therefore, the functional impairment of MFG and the left inferior frontal gyrus may be linked with decreased cognitive function post‐DBS.

There were several limitations in this study. First, the sample size was relatively small, and only a larger sample size could eliminate its impact on the experimental results. Second, although anti‐Parkinson's and other drugs were stopped before obtaining MRI data in this study, it is difficult to avoid the impact of levodopa accumulation on brain function. Finally, the 1.5 T field strength utilized in this study possesses a lower signal‐to‐noise ratio than 3.0 T. In the future, 3.0 T magnetic resonance helped verify the study results.

## 5 Conclusions

Our studies indicate that electrode implantation significantly affected three resting‐state functional networks associated with cognition (DMN, ECN, and DAN). Moreover, specific FC areas of DMN were associated with neurocognition in PD patients before DBS. This could help assess the cognitive level of PD patients and prepare them for cognitive rehabilitation after DBS. Further investigations are needed to identify network changes of cognitive decline after DBS.

## Author Contributions

All authors contributed to the study conception and design. B.L. and Y.X.Z. contributed equally to this work. Data collection and analysis were performed by B.L., Y.X.Z., X.W., J.S., Y.L., and W.W.D. Statistical analysis were performed by Y.X.Z., L.C., C.Q., and L.Z. The first draft of the manuscript was written by B.L., Y.X.Z., and J.Q.Y. W.B.Z., Y.H.Z., and J.Y. edited and revised the manuscript. All authors contributed to and approved the final manuscript.

## Conflicts of Interest

The authors declare no conflicts of interest.

## Data Availability

The relevant data used in this study are available from the corresponding authors upon request.
